# Location-specific deviant responses to object sequences in macaque inferior temporal cortex

**DOI:** 10.1038/s41598-024-54298-0

**Published:** 2024-02-14

**Authors:** Hamideh Esmailpour, Rufin Vogels

**Affiliations:** 1 Laboratorium Voor Neuro- en Psychofysiologie, Department of Neurosciences, KU Leuven, Leuven, Belgium; 2 Leuven Brain Institute, KU Leuven, Leuven, Belgium

**Keywords:** Extrastriate cortex, Object vision, Neuroscience, Psychology

## Abstract

Many species learn temporal regularities in their visual environment, demonstrating visual statistical learning. In this study, we explored the sensitivity of macaque inferior temporal (IT) cortical neurons to transition probabilities of sequentially presented visual images, presented at different locations in the visual field. We exposed monkeys to sequences of two images, where the first image was presented either foveally or peripherally, and the second image was consistently presented foveally. Following several weeks of exposure, we recorded IT responses to assess differences between the exposed (Fixed) and new, Deviant sequences, where the identity of the first image in a sequence differed from the exposure phase. While enhanced responses to Deviant sequences were observed when both images of a pair were foveally presented during exposure, no such deviant responses were present when the first image was presented peripherally. This finding challenges the notion that mere exposure to image sequences always leads to deviant responses in macaque IT. The results highlight the complexity of the mechanisms underlying statistical learning in primates, particularly in the context of peripheral image presentations, emphasizing the need for further investigation into the origins of these responses in the IT cortex.

## Introduction

Members of many species learn temporal regularities in their visual environment^1–4^, i.e., show visual statistical learning. The learning of transition probabilities of successively presented visual images affects the response of neurons in different parts of the brain in primates. Typically, exposure to a set of fixed sequences of visual images produces a smaller neural response to an image in its learned sequence compared to the response to that image when positioned in another sequence. This has been observed in the macaque inferior temporal (IT) cortex^5–12^, and frontal cortex^12^. A higher fMRI activation to stimuli that violated learned sequences, “deviants”, is also seen in various human brain regions, ranging from the early visual cortex^13–15^, over occipito-temporal cortex^14,16^ to regions outside the visual cortex such as the frontal cortex^14,15,17,18^. We will label here the enhanced response to the stimulus that violates the learned sequence as a deviant response. Note that this description remains agnostic to the possibility that it reflects an enhanced response with respect to a neutral condition (surprise enhancement) or a suppressed response to the expected stimulus in the learned sequence (expectation suppression^4,8,9,19^).

The origin of the deviant response in IT is still unclear. One possibility we recently tested is whether it reflects feedback from the prefrontal cortex that detects the violation of the learned sequence^12^. However, the deviant response onset was not earlier in the frontal cortex compared to IT, refuting this possibility. Here, we aimed to examine whether the deviant response reflects recurrent interactions within the ventral stream, as has been suggested by^11^. In that study, monkeys were exposed to pairs of faces that differed in head orientation. Responses of single units in the posterior IT face patch Middle Lateral (ML), which is selective for head orientation, to face pairs in which identity and orientation deviated from the exposed pair, showed an early deviant response to the violated identity and orientation, followed by a deviant response to only the violated identity, tolerating the change in face orientation. Since more anterior face patches show a greater tolerance to changes in head orientation than ML^20^, the late view-invariant suppression was interpreted as evidence for a view-invariant predictive signal from the anterior face patch to ML. This study suggests a role of recurrent interactions within the ventral visual stream in generating the deviant response. However, the early orientation-specific deviant response is in line with the idea that part of the deviant response reflects local processing^7^ or has a bottom-up origin.

We aimed to revisit this outstanding question by employing a strategy that is similar to the one employed before to assess the relative contribution of bottom-up input to adaptation in IT^21–23^. This strategy makes use of the changes in receptive field size along the visual hierarchy. Successive stimuli of a sequence are presented at different locations, and one assesses whether the effect under investigation generalizes across changes in location of the stimuli. When the effect generalizes across a change in location that is larger than the receptive field size of upstream areas then the effect is not inherited from those regions but is computed in the recorded area or reflects feedback.

In the present study, we adapted this location change paradigm to examine the contribution of bottom-up, local, and top-down processing to deviant responses in IT. We exposed monkeys to pairs of sequentially presented stimuli. For one set of pairs, the first stimulus of the sequence (S1) was presented ipsilateral to the recorded area while the second stimulus (S2) was presented foveally. We exposed the animals also to other stimulus pairs in which S1 was presented contralaterally and S2 foveally. Furthermore, we presented pairs in which both stimuli were presented foveally, as has been done in previous work^5^. After prolonged exposure to the different stimulus pairs during controlled fixation, we recorded multiunit activity (MUA) in IT to S1-S2 pairs for all possible combinations of S1 stimulus-location combinations, allowing us to examine the location generalization of the deviant response as a function of the visual field location of S1. We simultaneously recorded in both posterior and anterior IT. Posterior IT (PIT) neurons have smaller receptive fields than anterior IT (AIT) neurons^24,25^ and are expected not to respond or only weakly to the employed eccentric ipsilateral location (5 deg eccentricity^26,27^). Thus, a spatial generalization of the deviant response in PIT for sequences with the same S1 stimuli as were presented ipsilaterally during the initial exposure, in particular generalization to the contralateral S1 location, would suggest a top-down contribution to the deviant response from a region with larger and bilateral receptive fields like AIT.

This design allowed us to examine hypotheses of the contribution of bottom-up, local, and feedback to the deviant responses in PIT and AIT. However, the MUA showed the surprising result that the deviant response was present only for sequences in which S1 was presented foveally and that violated the exposed sequence in which S1 was also presented foveally. Neither in PIT nor in AIT, we observed consistent deviant responses following ipsilateral or contralateral S1 presentations when these were paired with foveal S2 presentations.

## Results

**Figure 1:**
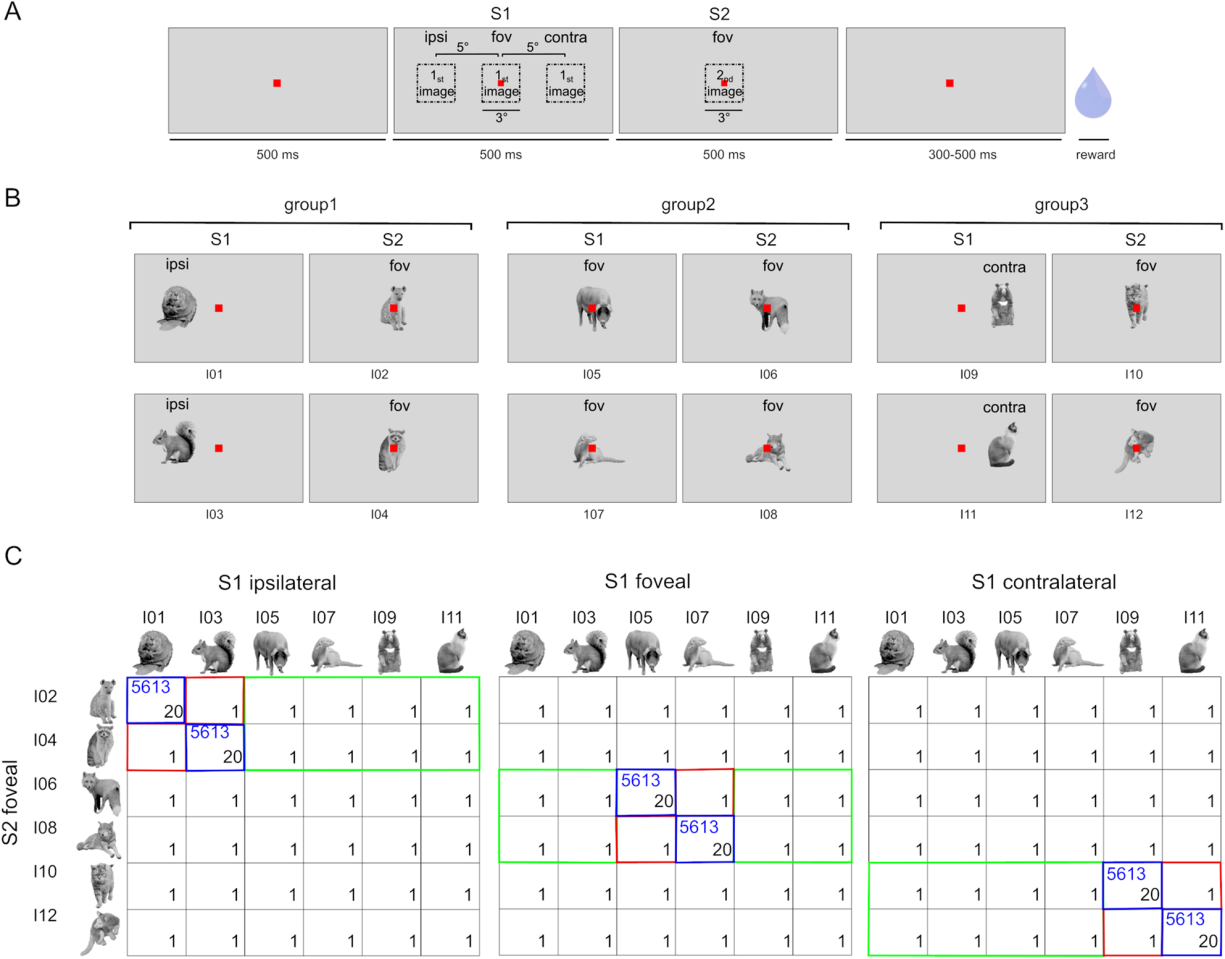
Experimental design and stimuli. (**A**) After fixation of a small square for 500ms, an image (S1) was presented for 500ms, followed by the second image (S2) presented for 500ms. The monkeys had to maintain their fixation for a variable duration (300–500 ms) after S2 offset to obtain a juice reward. The extent of the images was 3 deg. S1 was presented either at the center, at 5° eccentricity in the contralateral visual field, and at 5° eccentricity in the ipsilateral visual field. S2 was presented foveally.(**B**) Six pairs of images were presented to monkey M1 during the exposure phase. The order of images of a pair differed for M2. For the total of six pairs, S1 was presented at the ipsilateral position in group 1, at the foveal position in group 2, and the contralateral position in group 3. The frequency of occurrence during the total exposure phase for these pairs is indicated by the blue numbers in each panel of Fig. 1C. (**C**) The three panels show all the stimulus conditions of the recording phase presented in a single block. S1 included six different identities, which were labeled by odd numbers I01: I11, and S2 included six other identities labeled by even numbers I02: I12. Each panel shows a possible visual field location of S1. The number of trials per block for each condition is in black. The six pairs that were shown during the exposure phase occurred 20 times to avoid the extinction of learning and all the other conditions occurred once. Conditions shown by blue outlines were Fixed pairs, i.e., when the identities and location of S1 and S2 matched those from the exposure phase. Conditions shown by red outlines were Deviant pairs, i.e., when the identity of S1 differed from that of the corresponding Fixed sequence including the same S2 stimulus whereas its location was the same as in the exposure phase, and Deviant_position_change_S1, shown by the green outlines, i.e., when both the identity and location of S1 differed from the Fixed sequence with the same S2 identity.

**Figure 2:**
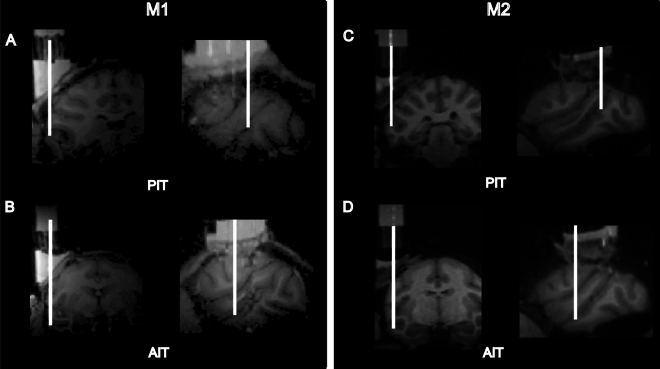
Recording locations in IT. Coronal and sagittal MRI slices of PIT and AIT of the brain of M1 (**A**, **B**) and M2 (**C**, **D**). The estimated location of an electrode penetration is indicated by a white line.

We exposed two monkeys to 6 pairs of successively presented images of animals during controlled fixation. For two pairs, the first stimulus of a pair (S1) was presented ipsilaterally at 5 deg eccentricity, for two other pairs S1 was presented foveally, and for the remaining pairs S1 was presented contralaterally (Fig. 1B). The second stimulus of a pair was always foveal. After exposure during many daily sessions, we recorded MUA simultaneously in PIT and AIT (Fig. 2). During the recordings, we presented all possible combinations of S1 identity-locations but with a strong bias for the exposed stimulus sequences (Fig. 1C) to avoid extinction of the learning.

**Figure 3:**
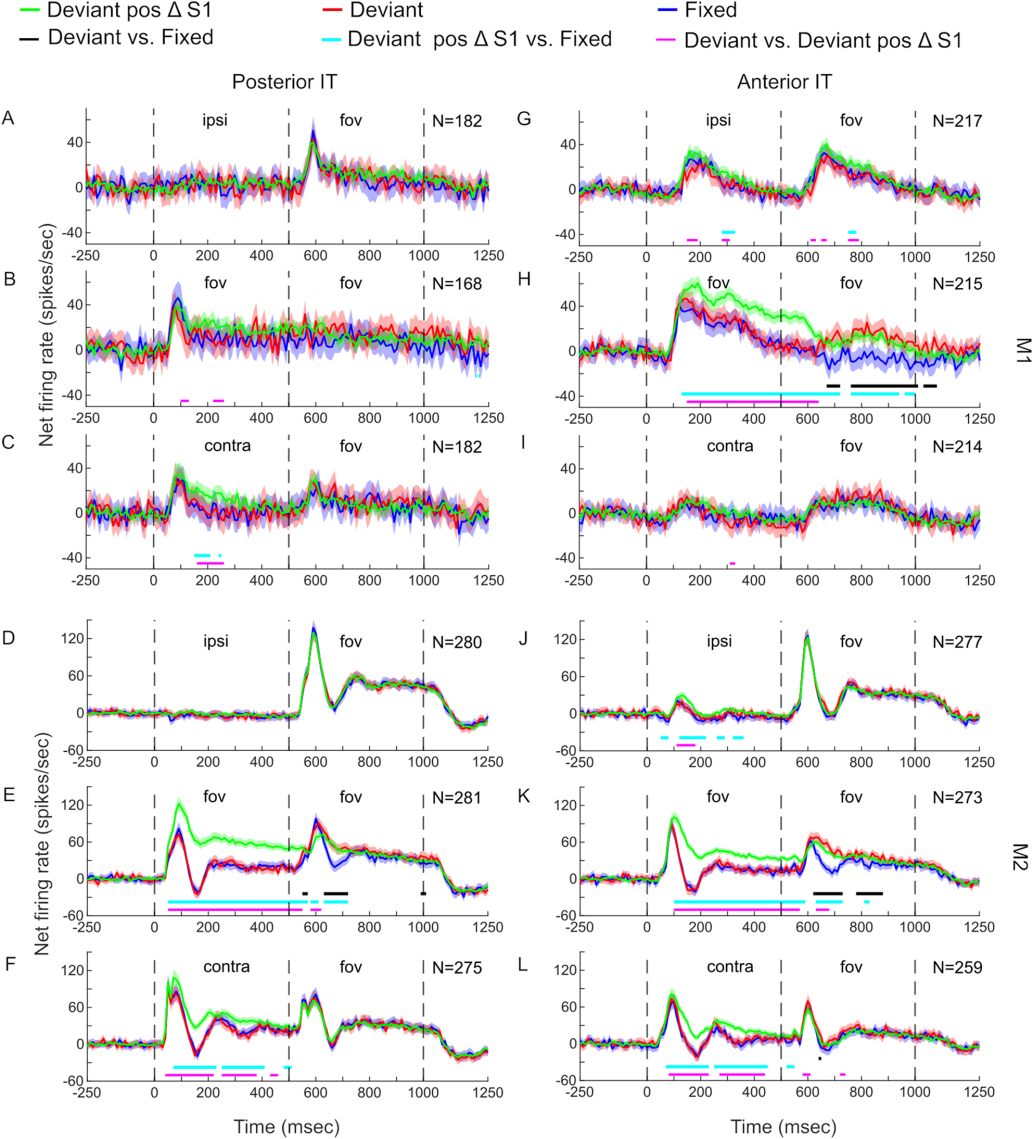
Average firing rate (MUA) to Fixed, Deviant, and Deviant_position_change_S1 pairs. The baseline response in the 250ms period before S1 onset (indicated by 0) was subtracted for each condition and unit before averaging the firing rate. The bin width was 10ms. S1 and S2 onset occurred at 0 and 500 ms, respectively. Bands indicate 95% confidence intervals. N corresponds to the number of units obtained in 29 and 36 sessions in M1 and M2, respectively. The averaged responses to image pairs of the Fixed sequences (blue boxes in Fig. 1C) are shown in blue and the responses to image pairs of the Deviant sequences (red boxes in Fig. 1C) are in red. The response to image pairs in which Deviant_position_change_S1 sequence (green boxes in Fig. 1C) are illustrated in green. The positions of the S1 and S2 stimuli are indicated above each PSTH (ipsi = ipsilateral; fov = foveal; contra = contralateral). The left column (A-F) and the right column (G-L) show population PSTHs of PIT (M1: A-C; M2: D-F) and AIT (M1: G-I; M2: J-L) for S1 locations at each peripheral and foveal position, respectively. The horizontal black lines indicate the clusters of 10 ms bins that show a significant response difference between Fixed and Deviant sequences, while the horizontal light blue lines present significant clusters for the Fixed versus Deviant_position_change_S1 sequences, and the horizontal pink lines show the significant clusters for the Deviant versus Deviant_position_change_S1 sequences (*p* < 0.05; corrected for multiple comparisons using the cluster-based permutation test; see Methods).

As expected, averaged PIT responses to S1 were present only when these stimuli were either foveal or contralateral (Fig. 3), in agreement with the smaller and largely unilateral receptive fields of PIT neurons. On average, AIT neurons showed bilateral responses.

To assess the presence of deviant responses related to a change of stimulus identity (different animal image), we compared the responses to S2 for the exposed stimulus pairs (Fixed sequences) and sequences in which the S1 image was at the same location as during the exposure phase but had the wrong identity (Deviant sequences; Fig. 1C, comparison of blue and red outlined stimuli). Note that the S1 and S2 stimuli were identical for these two sequence types, only the pairing differs. Because of that, we do not expect that the average response to S1 differed between the two sequence types, which was indeed the case for each monkey, IT region, and S1 location (red and blue curves in Fig. 3; Wilcoxon signed rank tests: all *p* values > 0.244 after FDR correction for multiple comparisons).

The responses to S2 were higher for the Deviant compared to the Fixed sequences in both IT regions and monkeys when S1 was presented foveally (Fig. 3B,E,H, and K). This deviant response was statistically significant when averaging the responses in a 500 ms analysis window, considering the S2 onset latency across conditions (see Methods; Wilcoxon signed rank tests: M1: PIT: *p* = 0.0357, AIT: *p* = 9.21e-15; M2: PIT: *p* = 7.43e-11, AIT: *p* = 5.24e-16). Cluster-based permutation tests using a bin width of 10 ms also showed significant differences between the two sequence types during S2 for the two monkeys and regions, except for PIT in M1 (Fig. 3).

Surprisingly, deviant responses were absent when S1 was presented in the periphery and thus S1 and S2 occupied different locations (Wilcoxon signed rank tests; all *p* values > 0.064; FDR corrected; Fig. 3), except for a small and brief deviant response in M2’s AIT when S1 was presented contralaterally (Fig. 3L; cluster-based permutation test). However, the latter was not significant when using the long analysis window (Wilcoxon signed rank test; *p* = 0.188; FDR corrected). Despite the absence of a deviant response, sizable S2 responses were observed in these sequences (Fig. 3).

Above, we compared the S2 responses for the same stimuli when these were preceded by S1 stimuli that had the same or a different identity as during the exposure phase and were presented at the same location as during the exposure phase. Next, we asked whether deviant responses would be present when the S1 stimulus differed in *both* location and identity from the exposed sequences. In this case, we compared the S2 response between the Fixed (blue outlined conditions in Fig. 1C) and Deviant_position_change_S1 (green outlined conditions in Fig. 1C) sequences. Note that the S1 images differed between the Fixed and the Deviant_position_change_S1 sequences and indeed the average response to the S1 responses differed between these two sequences for the contralateral and foveal S1 locations (Fig. 3). This difference in S1 response was significant for the foveal and contralateral conditions in each monkey for PIT (Wilcoxon signed rank tests; FDR corrected *p* values smaller than 0.004) and AIT (*p* values smaller than 1.53e-17), except for M1 AIT, contralateral (*p* = 0.40). For the ipsilateral conditions, a significant difference between these S1 responses was observed in the AIT of both monkeys (*p* values smaller than 0.045) but not in PIT (*p* values larger than 0.428), which is expected based on the differences in receptive fields between these two regions. It is noteworthy that the S1 responses, when different, were consistently higher for the Deviant_position_change_S1 compared to the Fixed condition (Fig. 3; green curves above blue (and red) curves). Note that the frequency of presentation did not differ between the S1 images across locations (Fig. 1C) but differed between locations for a particular image. Hence, the difference in S1 response between the two sequence types indicates a location-specific effect of presentation frequency, with a higher frequency resulting in less response at that location. Whether this is because of the exposure phase in which a particular image was shown only at one location, i.e., a location-specific familiarity effect, or because of the location bias during testing, i.e., a location-specific recency effect, is unclear.

The S2 responses for the Deviant_position_change_S1 sequences were similar to those observed for the Deviant sequences and this was for each of the S1 location conditions (Fig. 3). Thus, we observed a significantly greater S2 response for the Deviant_position_change_S1 sequences compared to the Fixed sequences in the foveal S1 condition in PIT (Wilcoxon signed rank test; M1: FDR corrected *p* = 0.0057; M2: *p* = 2.83e-06) and AIT (M1: *p* = 1.84e-13; M2: *p* = 1.2e-13). However, such a deviant response was absent when S1 was contralateral (*p* values larger than 0.437) and ipsilateral (*p* values larger than 0.064), except for a small but significant effect for M1 AIT (*p* = 0.023). The latter small effect could have resulted from the higher response to S1 in the Deviant_position_change_S1 sequence. Indeed, the interpretation of the greater S2 response to the Deviant_position_change_S1 compared to the Fixed sequences is tricky because of uncontrolled stimulus differences that can result in a difference in the response strength between the preceding S1 images. Nonetheless, the S2 responses to the former sequence were strikingly similar to those observed for the Deviant sequences, showing a sizable increased response relative to the Fixed condition only for the foveal S1 conditions.

We were concerned that the MUA of close sites of a probe might to some extent have been correlated which will have inflated the degrees of freedom in the statistical testing. To address this, we redid the statistical testing for the foveal condition after averaging the MUA of all sites for each penetration separately, so that each penetration (and not site) contributes one datum for the statistical testing. The deviant response observed for the foveal conditions remained significant in both monkeys for AIT (Wilcoxon signed rank test; M1: *p* = 7.60e-05; N = 29 penetrations; M2: *p* = 2.82e-06; N = 36) and in M2 for PIT (*p* = 1.51e-05). The smaller deviant effect in M1’s PIT did not survive this conservative testing (*p* = 0.09). Also, the greater S2 response to the Deviant_position_change_S1 compared to the Fixed sequences remained significant when averaging sites per penetration for AIT (M1: *p* = 1.19e-04; M2: *p* = 5.78e-06) and for M2 in PIT (*p* = 5.77e-05). Again, the smaller deviant trend in PIT’s M1 did not remain significant (*p* = 0.22; all *p* values FDR-corrected).

Because both Fixed and Deviant sequences differed between the two monkeys and each monkey showed a deviant response for the foveal conditions, the latter is unlikely a result from idiosyncratic image differences instead of a result from exposure, i.e., statistical learning. Nonetheless, we examined directly whether the across-monkeys consistent deviant response in the foveal conditions resulted from learning by comparing the difference in mean response between the 4 Fixed and 4 Deviant sequences with the mean response differences for each of 12 groups of 4 pairs of foveally presented sequences to which the monkeys were not exposed (Suppl. Fig. S1A). First, we pooled for each IT region the data of the two monkeys, after equating the number of sites for the two monkeys per region and computed the difference in mean response for the 4 Fixed (two per monkey) and 4 Deviant sequences of the foveal condition during the trial. The same analysis was performed for the responses to 12 different groups of 8 different unexposed sequences in which S1 and S2 were presented foveally and for which there was a response to the S2 images (see Methods for test of responsiveness). In each group, 4 sequences (two per monkey) were defined as “pseudo-Fixed” and 4 as “pseudo-Deviant” (light blue and red outlined conditions in Suppl. Fig. S1A). As for the real Fixed and Deviant sequences (dark blue and red outlined conditions in Suppl. Fig. S1A), the S1 and S2 images were identical for the corresponding pseudo-Fixed and pseudo-Deviant sequence pairs per monkey and group, the only difference between the sequence types being the pairing of the S1 and S2 images. Also, as for the real Fixed and Deviant sequences, the sequence pairs per group and monkey differed in their image order between the monkeys. Furthermore, four of the groups of unexposed sequences included images of the real Fixed and Deviant sequences. The number of trials per unexposed sequence was identical to that for the exposed sequences. We observed that the difference in mean response between the pseudo-Fixed and pseudo-Deviant sequences was smaller for *each* of the 12 sequence groups than the deviant response for the real Fixed and Deviant sequences (Suppl. Fig. S1B and C). This suggests that the deviant response in the foveal conditions resulted from statistical learning, and not from idiosyncratic image differences between sequences.

The absence of a deviant response for the ipsi and contralateral conditions did not result from an inability of the monkey’s brain to discriminate between the S1 images at 5 deg eccentricity. First, the difference in S1 response between the Deviant_position_change_S1 and Fixed conditions shows that the S1 images of these two conditions could be discriminated. The same regions that showed such a difference in the contralateral S1 response between these two sequences failed to show an enhanced response to deviant S2 stimuli (Fig. 3C,F). Second, we performed an additional analysis to show directly that the MUA showed selective responses to the S1 images of the Fixed and Deviant sequences, implying that these could be discriminated at 5 deg eccentricity. We employed a Wilcoxon rank sum test to select those MUA sites for which there was a significant difference in response (*p* < 0.05) between the two S1 images of those sequences when these were presented foveally during the recording. This we did separately for the two images that were presented either ipsilaterally or contralaterally during the exposure phase. Then, for each significant site, we ranked the two images as preferred or non-preferred based on their responses at the foveal location and applied that ranking to the S1 responses at the eccentric locations, followed by averaging the ranked S1 responses across sites for each of the eccentric locations, regions, and monkeys. We assessed whether, at the peripheral locations, the mean response was significantly greater for the preferred compared to the non-preferred ranked images (Wilcoxon signed rank tests; note that the ranking was based on the foveal location). This greater response for the preferred compared to the non-preferred rank was observed in M1 AIT at the ipsilateral (*p* = 0.015) and contralateral (*p* = 1.70e-04) location for the images that were presented ipsilaterally during the exposure phase and at both peripheral locations (*p* = 0.018; *p* = 3.22e-11) for the contralaterally presented images during the exposure phase. The same held for M2 AIT (all *p* values smaller than 8.76e-08). For PIT, trends were variable, reaching significance only in M2 at the contralateral locations for both the contra- and ipsilaterally presented images during the exposure phase (*p* values smaller than 3.15e-05). These results indicate not only that the MUA, especially in AIT, was sensitive to the difference between the images of the Deviant and Fixed sequences at the eccentric locations, but also that the image preference tolerated changes of location. The latter position tolerance of image preference in IT is expected based on a sizable literature^28–31^. In sum, the absence of a deviant response for the ipsi- and contralateral conditions did not result from an inability of the neurons to distinguish the S1 images at the employed eccentricity.

One possible factor that caused the absence of a deviant response for the peripheral conditions could be that it was somehow masked by the stronger response to the S2 stimuli when S1 was presented eccentrically compared to the foveal S1 conditions. To assess this possibility, we compared the S2 response for the same S1-S2 identity sequences of both peripheral conditions but when the S1 images were presented foveally (Suppl. Fig. S2A; light blue and orange outlines in the S1 foveal panel). However, no significant differences between the S2 responses for these sequences with foveal stimuli were observed in either IT region of M1, while there was a significant effect for the S2 responses corresponding to the contralateral sequences in M2 for PIT (Wilcoxon signed-rank test; *p* = 0.03; FDR corrected) and AIT (*p* = 3.19e-07; Suppl. Fig. S2B). However, the latter response differences between the S2 responses were numerically rather small compared to those for the sequences presented foveally during the exposure phase (compare foveal conditions of Fig. 3 and Suppl. Fig. S2B). Furthermore, the absence of an across-monkey consistent deviant response for the foveally presented sequences of which the S1 stimuli were presented contralaterally during the exposure phase raises doubt about the presence of a genuine deviant response for these peripheral conditions.

The presence of a deviant response for the sequences in which S1 was presented foveally during the exposure phase allowed us to examine whether this deviant response generalized across locations of the same S1 images. Thus, we compared the response to the S2 images that were preceded by a foveal S1 image during the exposure phase but now when these followed an ipsilateral or contralateral presentation of the same S1 image (light blue and orange outlines in S1 ipsilateral and S1 contralateral panels of Suppl. Fig. S2A; Fixed_position_change S1 and Deviant_position_change S1 conditions of Suppl. Fig. S2C). We did not find convincing evidence for spatial generalization of the deviant response: the contralateral S1 presentation sequences showed a significant effect in only AIT of only M2 (Wilcoxon signed-rank test; *p* = 0.0004; FDR corrected; Suppl. Fig. S2C), while all other examined sequences showed no significant deviant response. Furthermore, the significant deviant response for the contralateral S1 sequences in M2 was rather small compared to that observed when the same S1 images were presented foveally as during the exposure phase (compare foveal conditions of Fig. 3 with Suppl. Fig. S2C). Thus, unlike for the sequences in which S1 was presented foveally during the exposure phase, no across-monkey consistent deviant response to the same S2 images was observed when the S1 images of those sequences were presented peripherally after the exposure phase. The deviant response was restricted to foveal presentations of S1.

Since statistical learning can be reflected in the pupil response of monkeys^12,32^, we examined whether the mean pupil size differed between the Fixed and Deviant sequences for each of the 3 location conditions (Figure S3). In M1, mean pupil size was smaller for the Deviant S2 than for the Fixed S2 stimuli for the foveal S1 conditions (Wilcoxon signed-rank test; *p* = 0.039; uncorrected for multiple comparisons), which is in line with the pupil constriction observed before for Deviant stimuli of other learned sequences in this monkey^12^. However, this pupil size difference was no longer significant (*p* = 0.116) when correcting for multiple comparisons (three locations; FDR correction). No significant difference in pupil size for S2 between the two sequences was observed for the peripheral S1 location conditions (FDR corrected p’s larger than 0.178). M2 failed to show a statistically significant difference in the pupil size between the S2 stimuli for the two sequences at each S1 location (FDR corrected p’s larger than 0.221).

**Figure 4:**
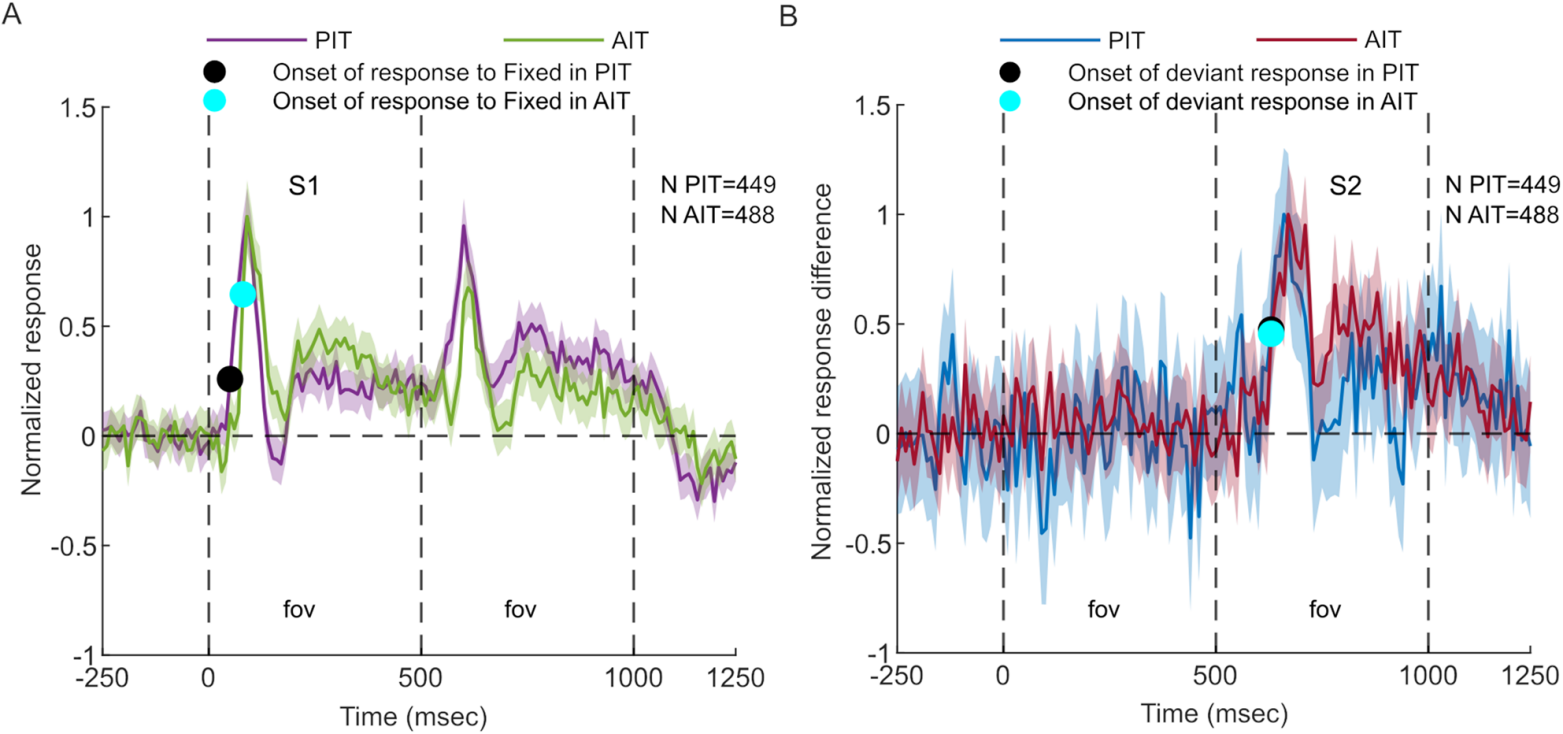
Differences in the response latencies between PIT and AIT. (**A**) Difference in response onset to the S1 stimulus of the Fixed sequences at the foveal position between PIT (purple) and AIT (green) for pooled data of M1 and M2. To facilitate the comparison of the latencies of the onset of the S1 response, the response was normalized by the maximum of the population response during the whole trial for each IT region individually. Bands indicate 95% confidence intervals of the mean response (bootstrapped across units). For each monkey, the dots indicate the estimated latencies for PIT and AIT, using the procedure described in the Methods. (**B**) Difference in response between the Deviant and Fixed sequences at the foveal position in PIT (dark blue) and AIT (dark red) for the pooled data of M1 and M2. The response difference was normalized by the maximum difference for each area. Bands indicate 95% confidence intervals of the mean difference (bootstrapped across units). The dots indicate the estimated latencies for PIT and AIT using the procedure described in the Methods. 0 corresponds to stimulus onset. The bin width is 10ms for all panels, and N shows the number of MUA units. S1 and S2 onset occurred at 0 and 500 ms, respectively.

The simultaneous recordings in PIT and AIT allowed us to assess whether the onset latency of the deviant response differed between the two IT regions. As expected, based on their position in the visual hierarchy, the onset latency of the S1 responses was significantly shorter in PIT compared to AIT (*p* = 0.005; bootstrap test; data pooled across the two monkeys; see Methods; Fig. 4A). However, the deviant response for the foveal conditions started at the same latency in PIT and AIT (Fig. 4B).

## Discussion

We exposed monkeys to sequences of image pairs in which the first image (S1) of a pair was located either foveally or peripherally and the second image (S2) was always presented foveally. After this exposure phase, lasting several weeks, we recorded in IT, assessing whether the response to S2 differed between the exposed (Fixed) and new, Deviant sequences in which the image identity of S1 of a pair differed from the one during the exposure. In agreement with previous studies from different groups^5–7,11,12^, we observed an enhanced response to Deviant compared to Fixed sequences in which both S1 and S2 were presented foveally during the exposure phase. However, no such across-monkey consistent deviant responses were observed for sequences in which S1 was presented peripherally during the exposure phase. This was true for sequences with ipsilateral or contralateral S1 presentations during the exposure phase. This finding suggests that mere exposure to image sequences does not always result in deviant responses in macaque IT, which has implications for the possible underlying mechanisms that drive these deviant responses.

Although the number of exposures to the image sequences was equal for the foveal and peripheral S1 conditions, we observed an across-monkey consistent deviant response for only the foveal conditions. Also, during the recording phase, the number of sequences in which the S1 images were paired with other S2 images than in the exposure phase was the same for the foveal and peripheral conditions. Thus, image statistics cannot explain the difference in the deviant response between the foveal and peripheral conditions.

One possible reason for the absence of a deviant response for the peripheral conditions is that the monkeys did not learn the predictive relations between the images when S1 is presented peripherally and S2 foveally. The pupil size analysis showed a difference between the Fixed and Deviant sequences in M1 (albeit not surviving multiple comparison correction) for the foveal but not peripheral conditions, which is in line with the IT recording data. However, M2 did not show a pupil size effect for any of the three location conditions. Thus, the pupil response data present ambiguity regarding statistical learning in our monkey subjects in the current study. However, the presence of deviant responses in IT during the foveal conditions indicates that at least the foveal condition sequences were learned by each monkey. Why then would the peripheral sequences not have been learned? The stimuli were presented during controlled fixation of a small foveal target, which can reduce attention to the peripheral stimuli. Statistical learning effects are reduced when subjects are performing an attention-demanding orthogonal task^16^. However, the onset of a peripheral stimulus will capture exogenous attention. It cannot be excluded though that such exogenous attention elicited by peripheral images is insufficient for statistical learning to occur even when an orthogonal attention-demanding task is absent, as during the passive fixation task in our experiment. To the best of our knowledge, in previous human studies of statistical learning in which stimulus location was manipulated^33,34^, eye fixation of the subjects was not controlled and hence stimuli might have been foveated, leaving open the question of whether visual statistical learning occurs for sequences of unfoveated stimuli.

It has been proposed that the relatively higher response to Deviant compared to Fixed sequences originates in IT by local Hebbian-like plasticity mechanisms: as a result of the repeated S1-S2 pairing, neurons that respond to the S1 image will inhibit more strongly neurons that respond to the repeatedly paired S2 image, resulting in a suppression of the activity specifically for the paired S2 image in the Fixed sequence^7^. Since IT neurons in the same hemisphere respond selectively to foveal and contralateral stimuli, one would expect that such a mechanism would result also in a suppression of the S2 response to the Fixed sequences when S1 was presented contralateral and S2 foveal during the exposure phase. However, this was not observed in the present experiment, which suggests that such a local plasticity mechanism, if present, is not as general as envisaged and at least requires revision.

We would like to note that our results do not show that statistical learning is not possible for peripheral stimuli when these are presented at the same (peripheral) location in the visual field. In our experiment, the peripheral S1 stimulus was shown at a different location than the S2 stimulus and this may have resulted in the absence of a statistical learning effect. Furthermore, our data also do not show that the statistical learning effect following exposure to stimulus sequences at the foveal location does not generalize to other, peripheral locations when both stimuli of the sequence are presented at the same peripheral location after the exposure. In fact, it has been reported that such spatial generalization of statistical learning-related responses for stimulus pairs in which the stimuli are presented at the same location occurs in IT^11^. We examined whether post-exposure spatial generalization of the deviant response observed for foveally presented sequences was present for the same image sequences when the S1 image was shown peripherally. We observed a weak effect for the contralateral S1 presentation condition only in the AIT of one of the two monkeys, unlike the much stronger across-monkey consistent effects seen in both monkeys when the same image sequence was presented foveally. Again, our post-exposure spatial generalization conditions included only sequences in which the S1 and S2 images were presented at different spatial locations, i.e. S1 peripheral and S2 foveal. Thus, it appears that the crucial factor determining visual statistical learning effects is not whether the stimuli are presented peripherally but whether both stimuli of the sequence are presented at the same or a different retinal location, but this conjecture needs to be examined in further experiments.

We observed a consistently higher response to S1 images that were presented peripherally and differed in location from the one during the exposure phase. This likely reflects the difference in presentation frequency of the images at the tested locations, with repeated exposure of a particular image at a particular location resulting in a reduced response. The presence of this location-specific stimulus familiarity or recency effect for the peripheral stimuli together with the absence of a statistical learning effect shows that effects related to stimulus frequency differ from statistical learning (or expectation suppression) effects. Related to this, it has been proposed that repetition suppression, i.e. the reduced response to a repeated stimulus^35,36^, reflects the same mechanism as expectation suppression, both resulting from a reduced response to an expected stimulus, with a repeated stimulus being an expected one^37^. We and others have shown that repetition suppression in IT partially transfers to other locations, i.e., is still present (although reduced) when S1 and S2 differ in location^21–23^. This contrasts with the present data in which expectation suppression (i.e., the deviant response) was observed consistently only when S1 and S2 were at the same location. This is consistent with the growing evidence that repetition suppression and expectation suppression rely on different mechanisms^4,38^.

Recently, we showed that the deviant response occurs later in the frontal cortex than in IT^12^, which is in line with a feedforward flow of prediction errors resulting from the mismatch between the predicted and presented stimulus in the deviant sequences. The present data show no evidence for a later onset of the deviant response in AIT compared to PIT, although the onset latencies for the S1 stimuli were shorter in PIT than in AIT. This conflicts with a feedforward flow of prediction errors between PIT and AIT. Because the deviant response in PIT of M1 was rather weak, this result is mainly due to the data of M2 and thus the absence of a difference in onset latency of the deviant response between the two regions should be taken with some caution. Nonetheless, it shows that the origin of the deviant responses in IT is still unclear and might result from a combination of factors. More generally, our data reveal that simply being exposed to temporally contiguous visual images is not sufficient to elicit responses related to transition probabilities in the macaque IT. One might wonder about the functional significance of the deviant responses in macaque IT when these are as specific to location changes of successive objects during exposure as was observed in the present experiment.

## Methods

The study is reported in accordance with ARRIVE guidelines. Sample sizes (number of animal subjects and number of penetrations) are based on previous studies of statistical learning effects in macaque IT^7,12^. 

### Subjects

Two female rhesus monkeys, M1 (7 kg; age: 12 years) and M2 (5 kg; age: 15 years) participated in this study. They were implanted with a magnetic resonance imaging (MRI)-compatible headpost for fixing the head during the experiments using standard procedures under full anesthesia (Propofol) and aseptic conditions. In addition, an MRI-compatible recording chamber targeted the posterior and anterior IT of one hemisphere. Animal care and experimental procedures complied with the regional (Flanders), European, and National Institute of Health guidelines and the experiments were approved by the Animal Ethical Committee of KU Leuven. One animal was euthanized with an overdose of Pentobarbital after this and other experiments. After completion of the experiments, we removed the implants of the second animal and she was accommodated in a zoo-like environment, socially housed with other monkeys.

### Recordings

Recordings were performed in the lower bank of the superior temporal sulcus (STS) of the anterior (AIT) and posterior IT (PIT) cortex (Fig. 2). Spiking activity from PIT and AIT was recorded simultaneously with two 8-channel V-probes (Plexon). The interspacing between channels was 150 µm with electrode sites linearly arranged on a single shaft. The V-Probes were lowered with two Narishige microdrives through two guide tubes that were fixed in a Crist grid. Recordings were made with a Plexon data acquisition system. Recorded signals were preamplified with a headstage having an input impedance of 1GΩ. Spiking activity was thresholded online using a threshold set at 2.5 standard deviations above the mean of each recording channel, and thresholded spike waveforms were saved at 40 kHz.

Eye position and pupil size were measured online with an infrared-based eye tracking system (ISCAN EC-240A, ISCAN; 120-Hz sampling rate). The analog eye movement and pupil signal were saved with a sampling frequency of 1 kHz. Eye positions, pupil size, stimulus, and behavioral events were stored for later offline analysis on a computer that was synchronized with the Plexon data acquisition system. The data was recorded from 4 grid positions in PIT and 2 grid positions in AIT in M1, as well as 2 grid positions in PIT and 3 grid positions in AIT in M2.

### Stimuli

Twelve gamma-corrected, achromatic images of animals were used as stimuli. The vertical or horizontal extent of each image was 3° of visual angle. The Shine toolbox was used to equate the mean luminance of each RGB channel across images. Stimuli were displayed on an LCD (Iiyama; 2560 × 1440 px; 1 ms GtG) on a gray background at a distance of 57 cm from the subjects’ eyes. The images were presented either in the middle of the screen or 5° to the left or right of the center. A small white square which was invisible to the monkeys was shown at the bottom left corner of the monitor together with the first frame of each image. A photodiode measured the onset of the square and was sampled at 40 kHz.

The 12 images were divided into six pairs of images. The order of the first and second images of each pair differed between the monkeys. The first image of a pair (S1) included 6 different identities (different animals) and the identity was labeled by odd numbers I01: I11. The second image of a pair (S2) included 6 other identities and its identity was labeled by even numbers I02: I12 (Fig. 1C). S1 could be shown ipsilaterally at an eccentricity of 5° on the horizontal meridian, at the center of the screen, or contralaterally at 5° on the horizontal meridian, while S2 was always shown at the center of the screen (Fig. 1A).

### Task

Each trial began with the appearance of a small red square, measuring 0.13 degrees in width, which functioned as the fixation target. Monkeys were required to keep their gaze steady at the center of the screen for a duration of 500ms (fixation window of 2.5° on a side). Subsequently, two images, S1 and S2, were presented consecutively, each lasting 500ms without any interstimulus interval. Following S2, the fixation target remained visible on the screen for a variable period, ranging from 300 to 500ms, before the delivery of a juice reward. The juice reward was given to the monkeys provided that they kept fixating throughout the whole trial (Fig. 1A).

### Exposure phase

For the exposure phase, six image pairs were presented in one block consisting of one presentation of each pair. The order of the pairs within a block was random. For two pairs, S1 was always shown at the ipsilateral position (Fig. 1B, group 1). For the two other pairs, S1 was shown at the foveal position (Fig. 1B, group 2), and for the two remaining pairs, S1 was shown at the contralateral position (Fig. 1B, group 3). Monkey M1 was exposed to the image pairs for 39 days, and monkey M2 was exposed for 65 days. The number of presentations of a pair was equated for the two monkeys, totaling 5613 for each image pair (shown in blue numbers in the paradigm matrix of Fig. 1C).

### Recording phase

In this phase of the experiment, one block consisted of 222 trials and 108 different conditions (Fig. 1C). The 108 image pairs consisted of all possible S1 identity (6) – location (3) combinations combined with each of the six S2 stimuli. These 108 conditions included the 6 Fixed pairs of the exposure phase. The order of presented pairs within a block was random. To counteract the extinction of the learning the Fixed pairs were presented 20 times each while the other pairs were presented only once per block (Fig. 1C). In addition, recording days were interleaved with training days in which only the 6 Fixed pairs were presented, i.e., the same as during the exposure phase. Therefore, during a week, 2 days were training days and 3 days were recording days. During a recording day, blocks were repeated until the animal stopped working.

### Image sequence types

Four types of image sequences were defined for the analysis of the recording phase data. When the identities and locations of S1 and S2 matched those from the exposure phase, it was categorized as Fixed. If the identity of S1 and S2 was the same as that of a Fixed sequence, but the location of S1 differed, the sequence was labeled as Fixed_position_change_S1. When the identity of S1 differed from that of a Fixed sequence whereas its location was the same, it was termed Deviant. Additionally, when both the identity and location of S1 differed from a Fixed sequence, it was labeled Deviant_position_change_S1. The comparison of responses to the same S2 identity between the sequences that included the same identities as in the Fixed sequence and the sequences in which S1 differed in identity from that in the Fixed sequence yielded what is referred to as the deviant response.

### Data analysis

#### Multiunit activity

Offline sorting of spikes was conducted to remove noise or electrical artifacts from the spiking activity, but no further spike sorting was performed. Thus, the spiking data reflected MUA. Only MUA site and block combinations that significantly modulated the MUA for S2 with respect to baseline were chosen for further investigation. For each trial, the average firing rate in a window starting 250ms before S1 onset was used to define the baseline activity. Additionally, the firing rate was computed during S2 presentation, using a window of 500ms duration with a 50ms delay after the S2 onset to account for the shortest response latency of IT neurons.

We tested separately the responsiveness for the three groups of S2 images irrespective of the position or identity of S1 (Fig. 1B). We conducted three split-plot ANOVAs for each block, using the baseline vs. analysis window as a within-trial factor and S2 identity as a between-trial factor. For further analysis, only blocks that demonstrated a significant baseline vs. stimulus activity main effect (*p* < 0.05) or a significant interaction between the two factors (*p* < 0.05) were taken into account. This method resulted in different numbers of sites for each group of pairs in PIT and AIT in M1 and M2 (Fig. 3). For the analyses of the deviant response, the number of trials was equated for the compared sequences by randomly selecting per block the same number of trials from the 20 trials of the Fixed condition. Net firing rates were computed by subtracting the baseline activity per site, and population peristimulus time histograms (PSTHs) were generated by computing the average net firing rates for each significant block of a given site and sequence type. Then, for each site we averaged the net firing rates across blocks, followed by averaging of these mean firing rates across the sites. Bootstrapping (MATLAB function "bootci"; bias-corrected and accelerated percentile method; resampling sites) was used to compute 95% confidence intervals (CIs) of the mean firing rate per 10ms bin. The difference between the mean firing rate for the Deviant and Fixed sequences was computed in bins of 10ms for each unit. The 95% CIs of the mean difference for each bin were obtained by bootstrapping sites.

#### Statistical comparison of responses

To assess whether the mean response differed significantly between sequence types, we computed for each site the mean net firing rate for S1 and S2 using two 500-ms analysis windows that started 130 ms after the onset of S1 and S2, respectively. The motivation behind selecting a 130 ms window for analysis was based on the observation that neurons exhibited a sustained response to a foveal S1 that persisted even beyond the initial 50 ms following the onset of S2. To ensure that the response to S1 did not confound the assessment of the response to S2, we determined the onset latency of the S2 response, averaged across Fixed, Deviant, and the Deviant_position_change_S1 sequences for each S1 location, per region and monkey. We found that the maximum response onset latency was 130 ms, explaining the use of this value as the delay time of the analysis window.

To test the pairwise difference in mean response between the sequence types we employed Wilcoxon signed-rank tests (number of observations being number of sites). The difference in mean response between Deviant and Fixed sequences was tested for S1 and S2 separately and corrected for multiple comparisons (false discovery rate (FDR)^39^: significance threshold q < 0.05). All these analyses were performed on the data of each monkey separately.

We used a cluster-based permutation test^40^ to assess the difference between the mean responses of a pair of sequences in 10ms bins. For each 10ms bin within the interval 0-1250ms post-S1 onset, we carried out a paired t-test of the difference between the firing rate of the two sequences and the absolute t-values of the significant bins (*p* < 0.05) were summed for each cluster of adjacent significant bins. We conducted 10,000 permutations by randomly shuffling the sequence type labels for each MUA site. We then applied the previously described method to generate summed absolute t-scores for the clusters and the highest sum was employed to create the null distribution. We determined the *p*-value for each original cluster as 100 – percentile of the original sum in the computed null distribution. We identified the original clusters as significant in cases where *p* < 0.05.

#### Computation of response latencies

We employed bootstrapping to test the significance of the difference in the S1 response onset for the Fixed sequences between PIT and AIT for the foveal position for the pooled data of the two monkeys. We resampled with replacement the units of the recorded PIT sample and the AIT sample, thus obtaining new PIT and AIT samples of the same size as the original samples. The latency of a region was quantified as the first 10ms bin of 4 successive bins for which the mean response (averaged across the units per sample) in the firing rate to S1 was greater than the mean plus twice the standard deviation (SD) of the binned mean response of the baseline period (0- 250 ms before S1 onset). The estimated latency of the response onset for the PIT units was subtracted from the AIT units. The resampling of the units was performed 1,000 times, resulting in 1,000 latency differences. The *p*-value corresponds to the percentile of zero (no latency difference) in the distribution of the latency differences, multiplied by two (two-sided test).

In addition, we computed the difference in latency of the deviant response onset for the foveal conditions between PIT and AIT. The difference between the firing rate for the Fixed and Deviant pairs, i.e., deviant response, was computed in bins of 10ms for each MUA site. The mean difference across all MUA sites was computed for each region separately and the latency of this mean difference response was computed for each region. The latency was quantified in the same way as was explained above, except for the duration of the baseline period, which was between − 250 and 500ms relative to S1 onset. We took the S1 responses also into account to compute the SD of the baseline differences since no difference in response between Deviant and Fixed pairs was expected (and was not present) for S1 (the deviant stimulus was S2). The estimated latency difference for the AIT units was subtracted from the latency of the deviant response of the PIT units. Since the latency difference between AIT and PIT was the same, no further statistical testing was performed.

### Pupil size

We compared the pupil size for the Fixed sequences and sequences in which the S1 image was at the same location as during the exposure phase but had the wrong identity (Deviant sequences; Fig. 1C, comparison of blue and red outlined pairs). The S1 and S2 stimuli were identical for these two sequence types, only their pairing differed. For each trial of these sequences, the pupil size data was smoothed with a 200-ms Hamming window. For each block, we equated the number of trials for the Deviant and Fixed pair trials by randomly selecting 2 trials for each of the two sequence types and each S1 location (ipsilateral, foveal and contralateral) separately. Then, we averaged the pupil size data for the 2 Deviant and the 2 randomly selected Fixed sequence trials, followed by subtraction of the mean baseline pupil size averaged in a 500 ms window that started 500 ms before S1 onset. This was done separately for each location. Then, we z-scored the baseline-subtracted mean pupil sizes after concatenating the Fixed and Deviant pairs for each location individually. Thus, we obtained for each block, z-scored pupil size measures for the Fixed and Deviant sequences and this separately for each location. These were averaged across blocks and sessions per subject and 95% confidence intervals of the mean z-scored pupil size were computed by bootstrapping blocks. To assess the statistical significance of the difference in pupil size between the deviant and fixed sequences for each location (ipsilateral, foveal and contralateral) and for each monkey, we averaged per block and sequence the z-scored pupil sizes in a 500-ms window that started 300 ms after the onset of S2. The 300-ms delay was employed because of the relatively long latency of pupil responses, compared to cortical neural responses, in cognitive tasks in macaques. Then, we employed for each location and monkey a Wilcoxon signed-rank test to assess the difference in S2 pupil size between the two sequences across blocks.

### Supplementary Information

Supplementary Figures.

## Data Availability

The datasets generated and analyzed during the current study are available at https://data.mendeley.com/preview/rn8bgzkvjj?a=134a944b-ee9f-4f52-84b6-7550f8c15984 and from the authors on reasonable request.
