# Supplementary material for: Location-specific deviant responses to object sequences in macaque inferior temporal cortex
Source: Sci Rep. 2024 Feb 14;14:3757. doi: 10.1038/s41598-024-54298-0 (PMC10866936; doi:10.1038/s41598-024-54298-0)
Supplement: Supplementary file 1 — Supplementary Figures. [file 41598_2024_54298_MOESM1_ESM.pdf]

Supplementary information

Location-specific deviant responses to object sequences in macaque inferior temporal cortex

Hamideh Esmailpour[1,2], and Rufin Vogels[1,2,*]

[1]Laboratorium voor Neuro- en Psychofysiologie, Department of Neurosciences, KU Leuven

[2]Leuven Brain Institute, KU Leuven, Belgium

[*] Corresponding author: rufin.vogels@kuleuven.be

Supplementary Figure 1.

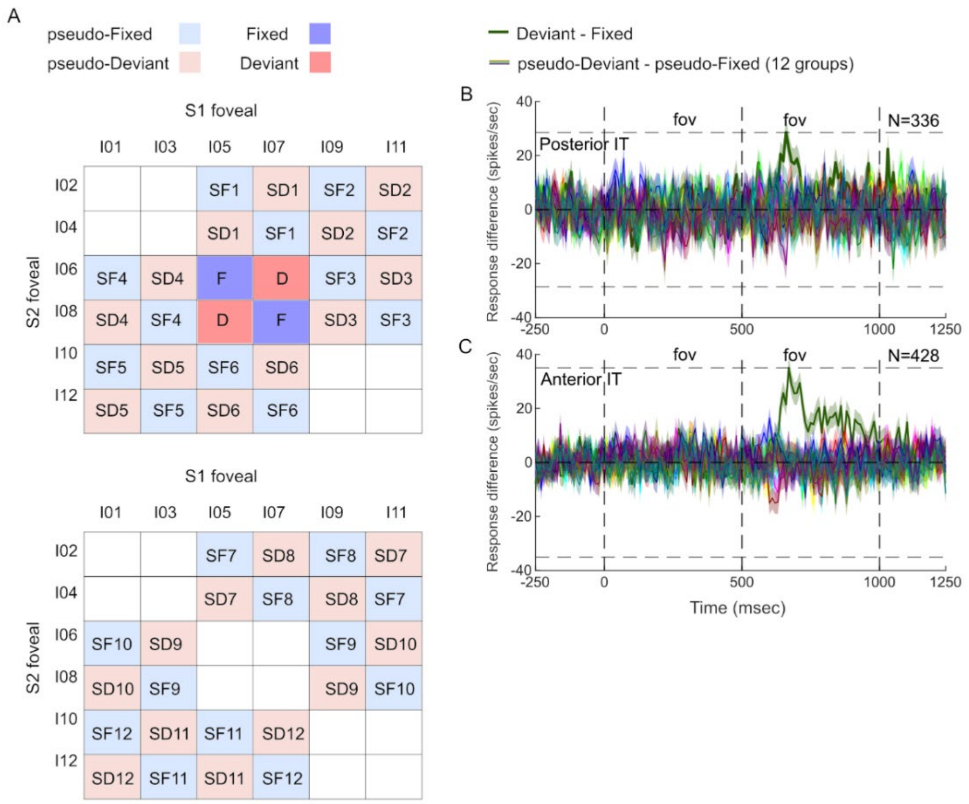

Supplementary Figure 1. Difference in firing rate between the Deviant and Fixed sequences compared to the response difference between the pseudo-Deviant and pseudo-Fixed sequences. A. *The dark blue and dark red filled squares indicate 2 groups of sequences of foveally presented S1 and S2 images, corresponding to the group of 2 Fixed image sequences (F; dark blue) shown foveally to the monkeys at the exposure phase (dark blue squares) and the groups of the 2 corresponding Deviant sequences (D; dark red squares). The other 12 groups correspond to 4 unexposed sequences each, in which S1 and S2 were presented foveally. Each of the 12 groups consists of 2 sequences that are defined as pseudo-Fixed (light blue squares; labeled by SFi, with i being a group) and 2 corresponding sequences defined as pseudo-Deviant (light red squares; labeled by SDi). For each IT region, the data of the two monkeys was pooled, resulting in 2 sequences per square in the panels.* ***B and C.*** *Comparing the difference in mean response between the Deviant and Fixed pairs (dark green line) with the difference in mean response between the pseudo-Fixed and pseudo-Deviant sequences for each of the 12 groups of unexposed sequences. The response difference for each group is indicated by a different color. B: PIT; C: AIT. Shaded bands indicate the standard error of the mean response difference for each group. N indicates the number of MUA sites. Dashed lines correspond to the maximum of the mean response difference for the Deviant versus Fixed comparison in each region.*

Supplementary Figure 2.

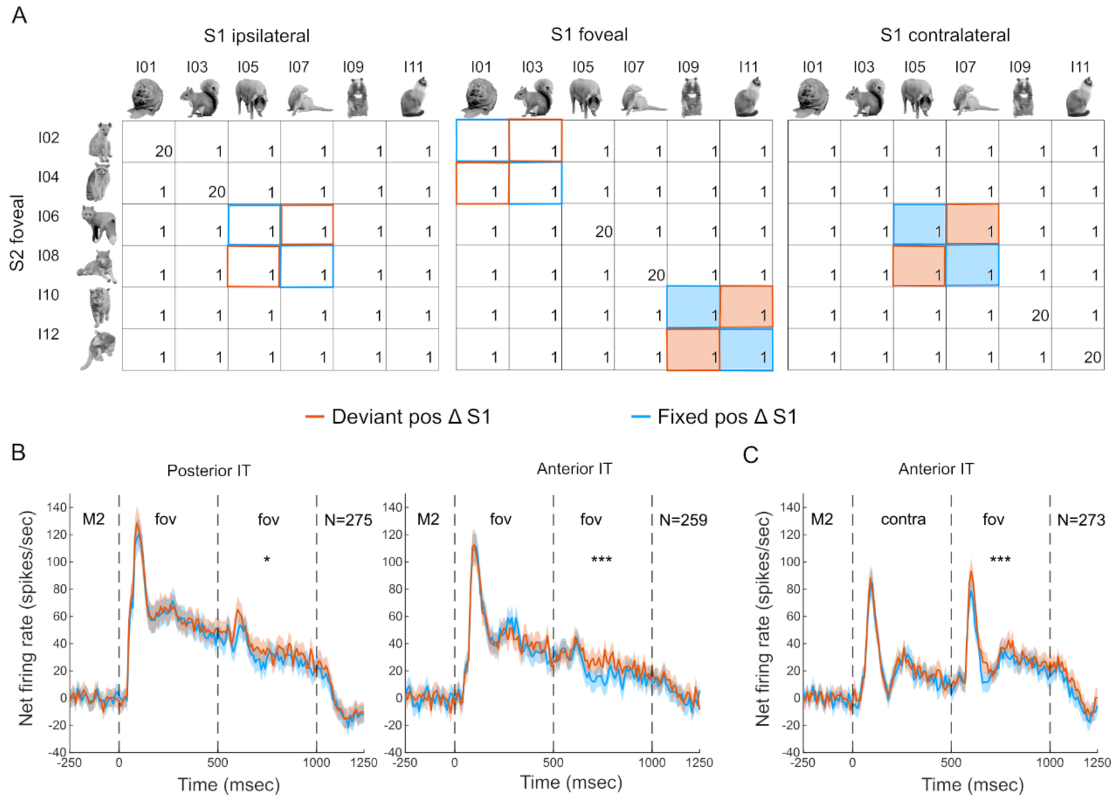

Supplementary Figure 2. Spatial generalization of deviant response. A. *"S1 ipsilateral" and "S1 contralateral" panels indicate in colored outlines the image pairs for which S1 was presented at a peripheral location and which have the same identities as the Fixed and Deviant image pairs presented foveally during the exposure phase. The light blue squares correspond to Fixed_position_change S1 and the light orange squares correspond to Deviant_position_change S1 sequences. The shaded squares in the "S1 contralateral" panel correspond to the sequences that showed a significant deviant response in AIT of M2 (see PSTH in panel C). The "S1 foveal" panel indicates in colored outlines the image pairs for which S1 was presented at a foveal location and which have the same identities as the Fixed and Deviant image pairs of which S1 was presented ipsilaterally and contralaterally during the exposure phase, respectively. Shaded squares in the "S1 foveal" panel correspond to the sequences that showed a significant deviant response in M2 (see PSTH in panel B).* ***B and C.*** *Comparison of responses to Deviant_position_change S1 and Fixed_position_change S1 in M2. The color codes of the conditions correspond to those of the colored outlines in panel A. Differences between conditions were tested by Wilcoxon-signed rank tests; FDR corrected for multiple comparisons. * p < 0.05; ** p < 0.01; **** p < 0.0001. N indicates the number of MUA units. M1 did not show significant response differences for these sequence comparisons. Note the numerically small effects in M2.*

Supplementary Figure 3.

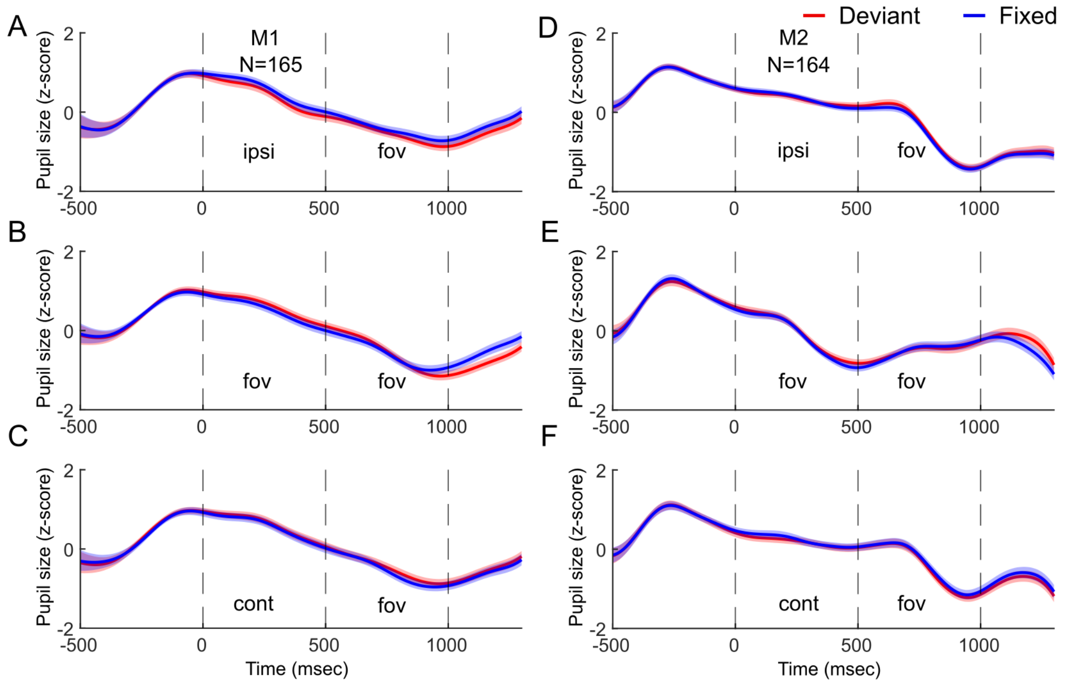

Supplementary Figure 3. Pupil responses. *Mean pupil size (z-scored; see Methods) for the Fixed (blue) and Deviant (red) sequences. Data were obtained during the IT recordings. Bands correspond to 95% confidence intervals. 0 and 500 corresponds to S1 and S2 onsets, respectively. N corresponds to number of blocks.* ***A-C****: pupil data of monkey M1;* ***D-E****: data of monkey M2.* ***A & D****: S1 at ipsilateral location;* ***B & E****: S1 at foveal location;* ***D & F****: S1 at contralateral location. Wilcoxon signed-rank tests showed no statistically significant difference between S2 of the two sequences that survived FDR correction for multiple comparisons (see Methods and Results for details).*
